# Small‐cell lung carcinoma and acute onset of antiglial nuclear antibody‐positive limbic encephalitis

**DOI:** 10.1002/ccr3.894

**Published:** 2017-03-08

**Authors:** Melvin Chan, Rajesh Rangaswamy, Yen‐Yi Peng

**Affiliations:** ^1^ University of Nevada‐Reno School of MedicineRenoNevadaUSA; ^2^ Radiology DepartmentRenown Institute for NeurosciencesRenown HealthRenoNevadaUSA; ^3^ Renown Institute for NeuroscienceRenown HealthRenoNevadaUSA; ^4^ Department of NeurologyUniversity of NevadaRenoNevadaUSA

**Keywords:** Antiglial nuclear antibody, limbic encephalitis, nonconvulsive seizure, small‐cell lung carcinoma

## Abstract

Limbic encephalitis (LE) can present as a nonspecific manifestation preceding neoplastic disease. Having high clinical suspicion and using newer onconeural antibodies, like antiglial nuclear antibody (AGNA), can lead to an earlier diagnosis. We report a patient with AGNA‐positive LE who is later diagnosed and treated for small‐cell lung carcinoma.

## Introduction

Limbic encephalitis (LE) presents with a myriad of symptoms that make the diagnosis challenging. These symptoms include seizures, amnesia, dementia, confusion, and psychosis. LE is believed to be a disorder affecting the medial temporal lobe of the brain. The origin can be from either paraneoplastic or autoimmune (nonparaneoplastic) causes. The diagnosis requires the following four criteria: an appropriate clinical presentation; ruling out of other oncological complications; <4 years between the development of neurological symptoms and the diagnosis of cancer; and one of the following findings: evidence of inflammatory changes in the cerebral spinal fluid (CSF), electroencephalogram (EEG) demonstrating abnormal electrical activity in the temporal lobes, or magnetic resonance imaging (MRI) showing structural abnormalities in the temporal lobes 1, 2. Assessing onconeural antibodies, oligoclonal bands, and protein levels in the CSF can assist in meeting the third and fourth criteria; positive results indicate evidence of inflammatory changes in the central nervous system, may raise the suspicion of an underlying paraneoplastic limbic encephalitis (PLE), and lead to further workup of occult cancer. One example is the association of PLE and testicular cancer for patients with anti‐Ma2 3.

A more recent discovery is the antiglial nuclear antibody (AGNA), which has a high positive predictive value for small‐cell lung cancer (SCLC), roughly 92% 4. This antibody was found through immunohistochemistry studies. Using DNA library screening studies, Sox‐1 was later found to react with AGNA in immunoblotting studies. Thus, AGNA and Sox‐1 antibodies are synonyms of each other 5. From a clinical standpoint, AGNA is more of cancer marker rather than a paraneoplastic syndrome (PNS) marker, because it can be found in cancer patients with or without neurological symptoms; other onconeural antibodies, such as anti‐Ma2 and anti‐Hu, are almost exclusively found in PNS 3, 4, 6.

This case report illustrates the usefulness of AGNA in prompting an early cancer workup for a patient, who presented with nonspecific gastrointestinal symptoms, which were later attributed to an early manifestation of limbic encephalitis. After this workup, the patient was given a diagnosis of SCLC within 3 months of her initial presentation.

## Case Report

This patient was a 70‐year‐old female presenting with an acute onset of intractable nausea and vomiting, mild epigastric pain, vertigo, generalized fatigue, and mild headache. Her past medical history was significant for type 2 diabetes, dyslipidemia, hypertension, and 23 pack‐years of smoking. She had no family medical history of neurological disorders. Physical examination was within normal limits except the following: amnesia (recalled 0/3 words). Despite this finding, the patient denied having any memory problems, and she remained alert and oriented throughout her first clinical encounter.

**Figure 1:**
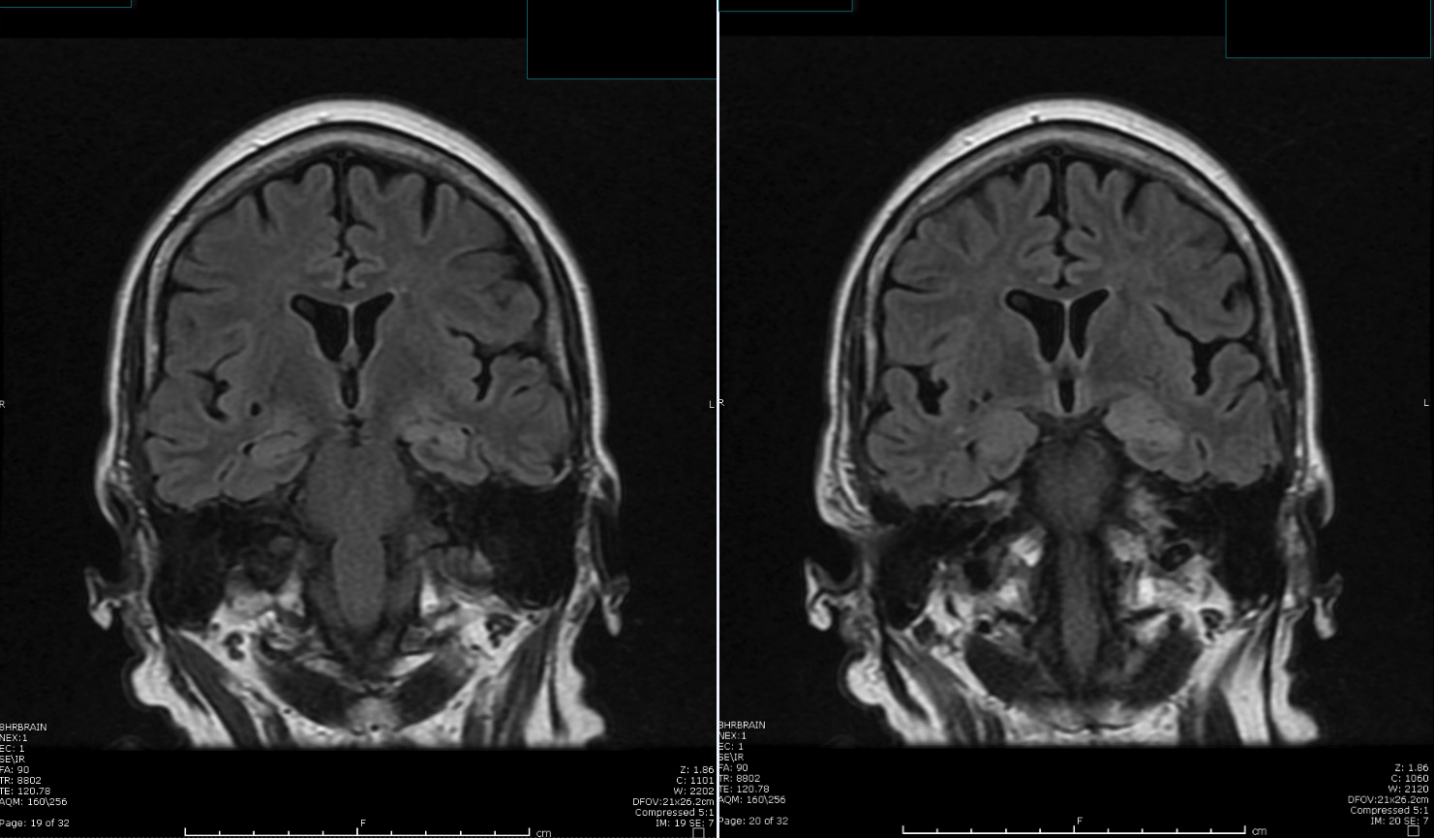
MRI of the brain showing subtle increased signal intensity on coronal FLAIR MRI sequences in both hippocampus (left > right).

A magnetic resonance imaging (MRI) was performed to evaluate her neurological symptoms. There was increased T2 signal intensity in the bilateral hippocampus on fluid‐attenuated inversion recovery (FLAIR) sequences, suggesting limbic encephalitis. Such a signal could easily be missed given its subtleness as seen in Figure 1, illustrating the importance of not over‐relying on the radiological report.

**Figure 2:**
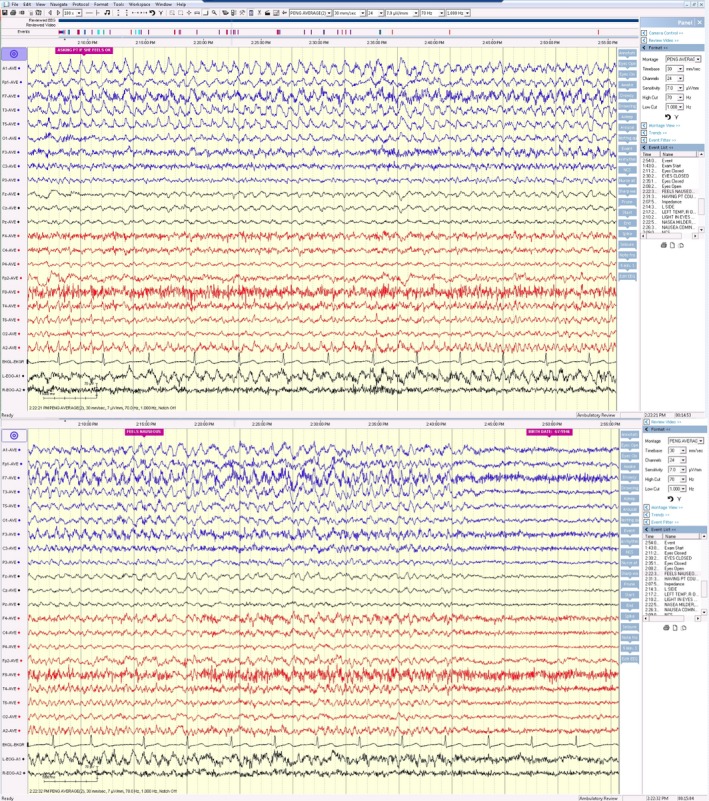
Video EEG showing rhythmic 4–5 Hz activity (maximum at F7, T3, and T5) with evolution of its amplitude, frequency, and morphology over the left hemisphere and subsequent spread to the right hemisphere, displayed on an average reference montage. These electrographic seizures corresponded well with the patient's report of nausea.

A video electroencephalogram (EEG) confirmed clusters of nonconvulsive seizures on the left hemisphere with spreading to the right hemisphere; each nonconvulsive electrographic seizure lasted for a minute and recurred every 5–10 min, as seen in Figure 2. The patient was able to associate these electrographic seizures with autonomic symptoms, such as nausea and vomiting 7.

A lumbar puncture was also performed, showing WBC 2 cells/*μ*L (reference range of 0–10 cells/*μ*L), RBC 5 cells/*μ*L (reference range of 0–1 cells/*μ*L), glucose 102 mg/dL (reference range of 40–80 mg/dL), total protein 38 mg/dL (reference range of 15–45 mg/dL), IgG 1.9 mg/dL (reference range of 0–6 mg/dL), and oligoclonal bands of 3 (reference range of 0–1 bands). There were no corresponding oligoclonal bands in the serum. Further testing of her CSF was negative for herpes simplex virus (HSV) DNA, Ebstein–Barr virus (EBV) DNA, Lyme antibodies, venereal disease research laboratory (VDRL) test, and Tropheryma whipplei polymerase chain reaction (PCR).

Additional analysis of her CSF indicated that her antiglial nuclear antibody (AGNA, or Sox‐1 antibody) had a high titer of 1:16 (the reference range of <1:2). These titer levels were determined using indirect immunofluorescence assay with conjugated goat antihuman IgG at Mayo Medical Laboratories 8. All of the following CSF tests through the Encephalopathy‐Autoimmune Panel of Mayo Medical Laboratories were also negative: NMDA‐R Ab CBA, VGKC‐complex Ab IPA, GABA‐B‐R Ab CBA, AMPA‐R Ab CBA, ANNA‐1 (antineuronal nuclear Ab, type 1), ANNA‐2 (antineuronal nuclear Ab, type 2), ANNA‐3 (antineuronal nuclear Ab, type 3), PCA‐1 (Purkinje cell cytoplasmic Ab, type 1), PCA‐2 (Purkinje cell cytoplasmic Ab, type 2), PCA‐Tr (Purkinje cell cytoplasmic Ab, type Tr), amphiphysin Ab, CRMP‐5‐IgG (collapsin response mediator protein 5), and GAD65 antibody.

Serum testing of antineutrophil cytoplasmic antibody IgG; Sjogren's anti‐SS‐A 52 (R0) IgG; Sjogren's anti‐SS‐A 60 (R0) IgG; Sjogren's anti‐SS‐B; antinuclear antibody; anticardiolipin IgA, IgM, and IgG antibodies; antithyroperoxidase (TPO) antibody came back negative.

Based on these findings, she met the criteria for limbic encephalitis. The patient was started on levetiracetam and solumedrol. Her nausea, vomiting, general weakness, and amnesia improved the following day. This symptomatic improvement coincided with a decrease in the number of nonconvulsing seizures during subsequent EEG monitoring. She was discharged after 7 days of hospitalization.

The differential diagnosis of limbic encephalitis included infectious encephalitis (including HSV, EBV. Lyme, and syphilis), low‐grade astrocytoma, gliomatosis cerebri, vasculitis, autoimmune disorders (including Sjogren's syndrome, lupus erythematosus, and Hashimoto's thyroiditis), and paraneoplastic causes. All of these causes except for paraneoplastic ones were ruled out with the extensive workup.

Positive AGNA typically correlates well with small‐cell lung carcinoma (SCLC) 3, 4, 6. Therefore, the patient had a whole‐body CT chest done, showing a mass‐like opacity in the anterior right upper lobe. Three months after her initial hospitalization, the patient underwent a right upper lobectomy, with the pathology reports confirming a 1.9 cm SCLC and negative ipsilateral hilar lymphadenopathy. These results further supported the case for paraneoplastic limbic encephalitis.

During a follow‐up visit, her family reported subjective improvements in the patient's memory and gastrointestinal symptoms. However, she admitted to being noncompliant with her antiseizure medication due to mild fatigue. After properly educating the patient and her family about the risk of noncompliance and the need to further titrate her dose to minimize adverse effects, she agreed to be more faithful to her treatment regimen. Further follow‐ups showed improvements in her memory. She was able to recall two of three words after 5 min on physical examination. Unfortunately, she refused chemotherapy and radiation and continued to smoke.

## Discussion

The case presented here is unique due to the use of antiglial nuclear antibody (AGNA) in the cerebral spinal fluid (CSF) in assisting with the diagnosis of small‐cell lung carcinoma (SCLC) in a patient presenting with an early manifestation of limbic encephalitis (LE), as shown in Figures 1 and 2. In addition to AGNA, other onconeural antibodies were tested in the CSF and found to be negative in the patient. Whether these antibodies were negative due to her early presentation of LE remains unknown. On the other hand, this case illustrates that AGNA can be positive in the early course of LE and is helpful in assisting with timely diagnosis of cancer. Table 1 lists common antibodies associated with LE and various carcinomas. These antibodies arise from a peripheral immune response against tumor antigens, which are structurally similar to other proteins in the central nervous system 3, 9, 10.

**Table 1:** Other onconeural antibodies seen in limbic encephalitis

Antibody	Associated conditions
Ma1/Ma2	Breast, Colon and Testicular (Ma1);Testicular Germ Cell Tumor (Ma2)
Cv2/Collapsing Response Mediator Protein‐5	Thymoma and SCLC
ANNA‐1 (anti‐Hu)	SCLC
ANNA‐2	SCLC, Breast Adenocarcinoma
ANNA‐3	Aerodigestive Carcinomas
PCA‐1	Adenocarcinoma of the Breast and Mullerian Ducts
PCA‐2	SCLC
PCA‐Tr/CRMP‐5 IgG	Hodgkin Lymphoma, SCLC, Thymoma
Amphiphysin IgG	SCLC and Breast Adenocarcinoma
GAD65	Tumors of Kidneys, Breast, Colon, and Thymus
GABA‐B	SCLC and Other Neuroendocrine Neoplasia
AMPAR	Lung, Breast, or Thymus Tumors
mGLuR5	Hodgkin Lymphoma
VGKC (or anti‐LG1)	SCLC, Thymoma, Adenocarcinoma of the Breast or Prostate
NMDA	Ovarian Teratoma

ANNA, Antineuronal antibody; PCA, Purkinje cell cytoplasmic antibody; GAD65, 65 kDa isoform of glutamic acid decarboxylase; GABA, Gamma amino butyric acid; AMPAR, Alpha‐amino‐3‐hydroxy‐5‐methyl‐4‐isoxazolepropionic acid receptor; mGluR5, Metabotropic glutamate receptor 5; VGKC, Voltage‐gated potassium channel; LG1, Leucine‐rich glioma inactivated 1; NMDA, N‐methyl‐D‐aspartate; SCLC, Small‐cell lung carcinoma.

This case also contributes to the current literature by correlating this patient's symptomatology (nausea and vomiting, mild epigastric pain, vertigo, general fatigue, mild headache, and amnesia) to the clusters of nonconvulsive seizures 11, 12. The patient reports that her electrographic seizures are associated with nausea, as seen in Figure 2. This patient has similar clinical findings to a 21‐year‐old patient with GAD65‐positive LE, who has been previously reported from this institution, including an acute onset of peri‐ictal autonomic gastrointestinal symptoms, amnesia, clusters of nonconvulsive seizures on video electroencephalogram, and increased T2 signal intensity in the bilateral hippocampus on fluid‐attenuated inversion recovery (FLAIR) sequences on magnetic resonance imaging (MRI). Both patients' symptoms have improved after optimization of their seizure medications and steroids, resulting in decreased seizure activity on follow‐up electroencephalogram. This clinical course supports the hypothesis that these nonconvulsive seizure clusters are the primary cause of memory problems, whereas the MRI abnormalities are best explained by postictal edema. The MRI images of other reported cases of limbic encephalitis vary due to the differences in the severity of seizure activity 9.

In conclusion, the diagnosis of LE can be difficult to make, especially early in its course and in patients with poor insight. A quick examination of a patient's memory needs to be undertaken with any neurological complaint, as it may be the only clue to the diagnosis of LE. Once suspected, MRI and EEG can facilitate formal diagnosis of LE. Afterward, a broad profile of onconeural antibodies in the CSF can assist in determining the etiology. Some of these antibodies, including AGNA, may be positive even early in the course of LE. Although these tests are often expensive, they can lead to a timely diagnosis of cancer and improve the odds of treatment.

## Conflict of Interests

All authors declare no conflict of interests.

## Authorship

MC: assisted in coordinating the patient's care with general surgery and wrote this manuscript. RR: was the neuroradiologist for this patient. YYP: was the primary neurologist for this patient.
